# Pollination, pollen tube growth, and fertilization independently contribute to fruit set and development in tomato

**DOI:** 10.3389/fpls.2023.1205816

**Published:** 2023-06-20

**Authors:** Long T. Tran, Koichi Sugimoto, Michael Kasozi, Oscar W. Mitalo, Hiroshi Ezura

**Affiliations:** ^1^Graduate School of Science and Technology, University of Tsukuba, Tsukuba, Japan; ^2^Faculty of Life and Environmental Sciences, University of Tsukuba, Tsukuba, Japan; ^3^Tsukuba-Plant Innovation Research Centre, University of Tsukuba, Tsukuba, Japan

**Keywords:** x-ray irradiated pollen, pollen tube growth, parthenocarpy, tomato fruit set, tomato fruit development

## Abstract

In flowering plants, pollination, pollen tube growth, and fertilization are regarded as the first hierarchical processes of producing offspring. However, their independent contributions to fruit set and development remain unclear. In this study, we examined the effect of three different types of pollen, intact pollen (IP), soft X-ray-treated pollen (XP) and dead pollen (DP), on pollen tube growth, fruit development and gene expression in “Micro-Tom” tomato. Normal germination and pollen tube growth were observed in flowers pollinated with IP; pollen tubes started to penetrate the ovary at 9 h after pollination, and full penetration was achieved after 24 h (IP24h), resulting in ~94% fruit set. At earlier time points (3 and 6 h after pollination; IP3h and IP6h, respectively), pollen tubes were still in the style, and no fruit set was observed. Flowers pollinated with XP followed by style removal after 24 h (XP24h) also demonstrated regular pollen tubes and produced parthenocarpic fruits with ~78% fruit set. As expected, DP could not germinate and failed to activate fruit formation. Histological analysis of the ovary at 2 days after anthesis (DAA) revealed that IP and XP comparably increased cell layers and cell size; however, mature fruits derived from XP were significantly smaller than those derived from IP. Furthermore, there was a high correlation between seed number and fruit size in fruit derived from IP, illustrating the crucial role of fertilization in the latter stages of fruit development. RNA-Seq analysis was carried out in ovaries derived from IP6h, IP24h, XP24h and DP24h in comparison with emasculated and unpollinated ovaries (E) at 2 DAA. The results revealed that 65 genes were differentially expressed (DE) in IP6h ovaries; these genes were closely associated with cell cycle dormancy release pathways. Conversely, 5062 and 4383 DE genes were obtained in IP24h and XP24h ovaries, respectively; top enriched terms were mostly associated with cell division and expansion in addition to the ‘plant hormone signal transduction’ pathway. These findings indicate that full penetration of pollen tubes can initiate fruit set and development independently of fertilization, most likely by activating the expression of genes regulating cell division and expansion.

## 1 Introduction

Tomato (*Solanum lycopersicum* L.) is both an economically important crop in the world and a model plant for fruit science and production (Ezura, 2009). Fruit initiation and development from tomato flowers can be divided into four distinct phases (Ariizumi et al., 2013; Quinet et al., 2019), viz. fruit set (phase I), cell division (phase II), cell expansion (phase III), and fruit ripening (phase IV), all of which require the coregulation of genetic and hormonal elements *via* complicated pathways (Molesini et al., 2020; Fenn and Giovannoni, 2021). Both pollination and fertilization are believed to be prerequisites for fruit set and development (Quinet et al., 2019), but seedless fruits can be produced independently of fertilization, as is the case with the parthenocarpy phenomenon which can be achieved either by exogenous hormone treatments or genetic mutation approaches. Parthenocarpy is a highly desirable agronomic trait, as fruit formation is less affected by environmental factors (Molesini et al., 2020). Thus far, several parthenocarpic mutations, such as *pat-2*, *iaa9-3*, and *pad-1*, display high seedless fruit set ratios, and hence, they are considered potential genetic materials for the breeding of seedless tomato fruit cultivars (Takisawa et al., 2019; Matsuo et al., 2020; Takisawa et al., 2020; Tran et al., 2021).

Like in other flowering plants, pollination in tomato occurs on the stigma surface as the first step in the reproduction process. Pollination is then followed by germination of pollen grains to form unique structures known as pollen tubes. The pollen tubes provide a link between pollination and fertilization, as they act as vehicles to deliver sperm cells in pollen grains to egg cells in the ovules which are located in the ovary. Interestingly, soft X-ray-irradiated pollen containing inactivated sperm cells produced standard pollen tubes which penetrated ovules and eventually resulted in parthenocarpic fruit development in watermelons (Hu et al., 2019). The soft X-ray-irradiated pollen induced both auxin signalling and the accumulation of various hormones including gibberellins, cytokinins, and auxins, and the resultant parthenocarpic watermelons were comparable in size to normal seeded fruits (Hu et al., 2019). Other reports have also demonstrated that there is a swift activation of ethylene biosynthesis and perception during pollen tube growth in multiple plant species (Holden et al., 2003; Jia et al., 2018; Althiab-Almasaud et al., 2021). These data suggest the potential roles of pollen tubes in the regulation of hormonal synthesis and signalling to induce fruit set and development even in the absence of fertilization. In tomato, however, soft X-ray irradiation applied directly to dried pollen strongly impaired pollen germination and led to production of tiny parthenocarpic fruits (Nishiyama and Tsukuda, 1961; Uematsu and Nishiyama, 1967). In this sense, no further research on this topic has been conducted since then, and hence the potential use of X-ray-irradiated pollen to produce parthenocarpic fruits in tomato remains unexplored. Furthermore, the role of pollen tubes in fruit initiation and development at the molecular level in tomato is still unknown.

In this study, we used dead, intact, and soft X-ray-treated pollen to explore the independent effects of pollination, pollen tube growth, and fertilization on fruit initiation and development in tomato. Partial pollen tube growth in the styles triggered the expression of various genes which are associated with the release of cell cycle dormancy, but these changes did not adequately initiate fruit set. However, full penetration of the pollen tubes into the ovary activated genes associated with cell expansion and division most likely through many hormonal pathways independently of fertilization and eventually initiated fruit set and development. In addition, we show that fertilization could contribute to the latter stages of fruit development by activating the expression of a distinct set of cell expansion genes. Altogether, these findings suggest that pollen tube penetration into the ovary can sufficiently trigger normal fruit set and development regardless of fertilization, a physiological function of pollen tubes that has not been established previously in tomato.

## 2 Materials and methods

### 2.1 Plant material and growth conditions

Seeds of *S*. *lycopersicum* cultivar “Micro-Tom”, both wild type (ID: TOMJPF00001) and an EMS parthenocarpic mutant *iaa9-3* (ID: TOMJPE2811-1), were supplied by the National Bioresource Project archived at the TOMATOMA database^1^. The plants were grown in rockwool blocks in a growth room set at 25°C under photosynthetically active light (75-110 mmol/m^2^/s) for 16 h and 20°C in the dark for 8 h.

### 2.2 Pollen preparation

Fresh anther cones were collected at the anthesis stage and then used to prepare three types of pollen, that is, dead pollen (DP), soft X-ray-treated pollen (XP), and intact/normal pollen (IP). To prepare DP, the anther cones were dried at 100°C for 2 h. IP were prepared by drying the anther cones at 35°C for 6 h. Finally, to make XP, fresh anther cones were first subjected to a soft X-ray irradiation (Model: OM-303M, OMIC Corporation, Japan) of 1000 Gy for 72.15 minutes followed by drying at 35°C for 6 h. The protocol for soft X-ray irradiation was derived from our work (unpublished data) at the University of Tsukuba.

### 2.3 Pollination and treatments

Flowers were emasculated one day before anthesis (-1 DAA) to avoid self-pollination. The emasculated flowers were then pollinated on the next day (0 DAA) using the pollen types described in section 2.2. Styles were removed from IP-pollinated flowers 3 h, 6 h, 9 h, 12 h, and 24 h after pollination (denoted as IP3h, IP6h, IP9h, IP12h, and IP24h, respectively). For XP- and DP-pollinated flowers, styles were removed 24 h after pollination (denoted as XP24h and DP24h, respectively). In all cases (IP3h, IP6h, IP9h, IP12h, IP24h, XP24h and DP24h), ovaries that remained after style removal were left to stand on the plants (10 plants per each treatment) for further histological and RNA-Seq analyses as well as assessment of fruit set ratios. The ovaries were collected at 2 DAA (for RNA-Seq analysis) and from 2–10 DAA (for histological analysis). Ovaries from emasculated but unpollinated flowers (denoted as E) with style removal 24 h after anthesis were also collected at the same timepoints to represent control treatments.

### 2.4 *In vivo* pollen tube growth assays and aniline blue staining

Pistils were collected 3 h, 6 h, 9 h, 12 h, and 24 h after pollination with IP pollen, and 24 h after pollination with XP and DP. The pistils were then fixed in 3:1 ethanol (100%): acetic acid (100%) solution for 12 h, washed in 70% ethanol and finally softened in 5 N NaOH for 12 h. Softened pistils were washed five times in distilled water and stained overnight in the dark with 0.01% aniline blue solution in K_3_PO_4_ buffer. The stained pistils were then mounted in 100% glycerol on a slide and observed under a UV microscope (Olympus BX50, Olympus-Life Science Company, Japan). At least three pistils were observed for each of the seven treatments.

### 2.5 Determination of fruit set ratios

Fruit set ratios were evaluated using five different plants and a total of 21-30 flowers for each style removal (IP3h, IP6h, IP9h, IP12h, IP24h, XP24h and DP24h) treatment. The ratios were expressed as the percentage of ovaries which remained on the flowers and showed an increase in size at 10 DAA.

### 2.6 Histological analysis and fruit growth measurements

Histological analyses were carried out using Technovit 7100 (Kulzer Technik, Germany) according to the protocol of Yeung and Chan (2015) with slight modifications. Briefly, ovaries collected at 0, 2, 4, and 10 DAA were fixed as described in Section 2.4 and then dehydrated by passing them sequentially through graded ethanol (70%/2 h → 80%/2 h → 90%/2 h → 100%/overnight). This was followed by three infiltration steps in ethanol:Technovit solutions at different concentrations (2:1/2 h → 1:1/2 h → 1:2/2 h), and overnight immersion in pure Technovit. The ovaries were then embedded in a mixture of Technovit and Hardener II (15:1 v/v) and allowed to polymerize at room temperature for 12 h before sectioning at a thickness of 5 μm using a rotary microtome machine (Leica RM2235, Leica Biosystem Ltd., China). Finally, the sections were mounted in water on a glass slide, dried at 40°C, and stained with toluidine blue (Sigma-Aldrich T3260, Merck, USA). A drop of Entellan New (Sigma-Aldrich 107961, Merck, USA) was added to the slide before observation under a light microscope (Olympus BX50). At least three ovaries were observed for each style removal treatment. To estimate fruit growth, cell layer and cell volume measurements were conducted on the microscope images using ImageJ software.

### 2.7 RNA extraction, library construction and RNA sequencing

Samples for RNA-Seq analysis included emasculated but unpollinated ovaries (E) as a control (absence of pollination, pollen tube growth and fertilization), and ovaries from IP6h, IP24h, DP24h, and XP24h style removal treatments. These samples were selected based on pollen tube growth observations; IP6h represented partial pollen tube growth (no penetration into the ovaries and hence no fertilization), IP24h represented full pollen penetration into the ovaries with fertilization, XP24h represented full pollen tube penetration without fertilization, while DP24h represented pollination only (no pollen tube growth and no fertilization). All samples were collected 48 h after pollination (2 DAA), and each sample contained 5 ovaries with three replications. Total RNA was extracted from the ovary samples using the RNeasy^®^ Plant Mini Kit (Qiagen, Hilden, Germany) according to the manufacturer’s instructions. Treatment with DNase I (Nippon Gene, Tokyo, Japan) was carried out to remove genomic DNA contamination. mRNA was then purified from the total RNA using a poly(A) mRNA magnetic isolation module kit (New England BioLabs). The pure mRNA was used to construct paired-end libraries for Illumina using the Ultra™ II directional RNA library prep kit (New England BioLabs), and sequencing was performed on an Illumina NovaSeq 6000 platform (Illumina, Inc.).

### 2.8 RNA-seq data analysis

RNA-Seq data were analysed on the Galaxy main server^2^. The raw RNA sequence reads were qualified using the FastQC tool and then trimmed by the Trimmomatic tool (Bolger et al., 2014). The resultant high-quality sequences were then mapped to tomato reference genomes (v. SL4.0) using the Hisat2 tool with default parameters (Kim et al., 2019). Mapped reads were counted using the featureCounts tool (Liao et al., 2014). The read counts obtained were normalized to the gene expression level as transcripts per kilobase million (TPM) reads. Gene Ontology (GO) enrichment and Kyoto Encyclopedia of Genes and Genomes (KEGG) analyses were conducted on the web application iDEP (v. 0.93)^3^ and ShinyGO (v. 0.77)^4^ (Ge et al., 2018).

### 2.9 Statistical analysis

Data obtained in this study are presented as average values ± SE. Mean comparisons were tested by Tukey’s HSD (honestly significant difference) test at *P* < 0.05. All statistical analyses were performed using Statistical Tool for Agricultural Research (STAR), version 2.0.1 (Gulles et al., 2014).

## 3 Results

### 3.1 Effect of pollen tube growth on fruit setting in tomato

**Figure 1:**
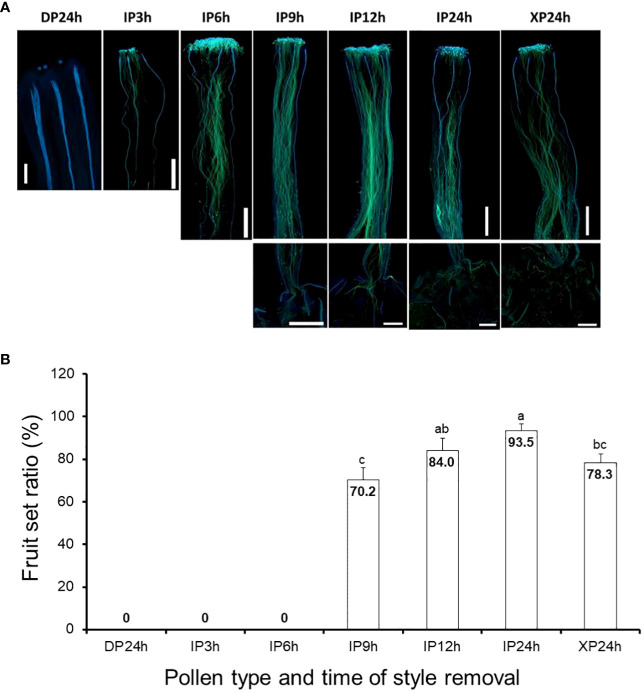
Pollen tube growth and its effect on fruit setting in tomato. **(A)** Images showing pollen tubes in the pistils. Pollen tubes were stained by aniline blue and appear green under UV microscopy at a magnification of 40X (n = 6). Lower panels in IP9h, IP12h, IP24h and XP24h columns indicate pollen tube signals inside the ovary. **(B)** Fruit set ratios of ovaries of the specified pollen type and style removal treatments. Values are percentages of fruit numbers on the examined flowers (21–30 flowers per treatment) at 10 DAA. Different letters indicate statistical difference using Tukey’s HSD (honestly significant difference) test at *P* < 0.05. White bars in **(A)** images = 50 μm. IP3h – pollinated with intact pollen and styles removed 3 h later; IP6h – pollinated with intact pollen and styles removed 6 h later; IP9h – pollinated with intact pollen and styles removed 9 h later; IP12h – pollinated with intact pollen and styles removed 12 h later; IP24h – pollinated with intact pollen and styles removed 24 h later; XP24h – pollinated with x-ray-irradiated pollen and styles removed 24 h later; DP24h – pollinated with dead pollen and styles removed 24 h later.

*In vivo* pollen tube growth assays, following pollination of tomato flowers with IP, XP and DP, were crucial in this study to determine the independent effects of pollination, pollen tubes, and fertilization on fruit setting. For IP-pollinated flowers, pollen tubes elongated to approximately one-third and three-quarters of the styles after 3 h and 6 h (**Figure 1A**), respectively and the earliest penetration of the pollen tubes into the ovaries, indicated by a limited signal below the style baseline, was observed 9 h after pollination. Full penetration of IP-generated pollen tubes into the ovaries occurred 12 h and 24 h after pollination, as the signal strengths inside the ovaries were comparable. Likewise, pollen tubes in XP-pollinated flowers elongated and fully penetrated the ovaries 24 h after pollination (**Figure 1A**). As expected, DP failed to germinate and hence pollen tubes could not be observed in the styles of DP-pollinated flowers even after 24 h. Therefore, at the specified times of style removal, pollen tubes were still in the styles for IP3h and IP6h, but there was partial penetration into the ovaries for IP9h. For IP12h, IP24h and XP24h, full penetration of the pollen tubes into the ovaries had already taken place.

Ovaries that were left on the plants after style removal treatments were then assessed at 10 DAA for their ability to develop into fruits. Results indicated that IP3h and IP6h ovaries failed to initiate fruit set, similar to DP24h (**Figure 1B**). On the other hand, IP9h ovaries displayed a considerable fruit set ratio (70.2%), although it was significantly lower than IP12h (84.4%) and IP24h (93.5%) ovaries. Interestingly, XP24h ovaries also showed a noticeably high fruit set ratio (78.3%), albeit significantly lower than that of IP24h ovaries. Together, these findings indicate that pollination alone (represented by DP24h) or partial pollen tube growth (represented by IP3h, IP6h and to some extent, IP9h) were not sufficient to initiate fruit setting. Contrarily, full penetration of pollen tubes into the ovaries even in the absence of fertilization (as shown in XP24h) can sufficiently trigger fruit set.

### 3.2 Full penetration pollen tubes results in normal fruit development

**Figure 2:**
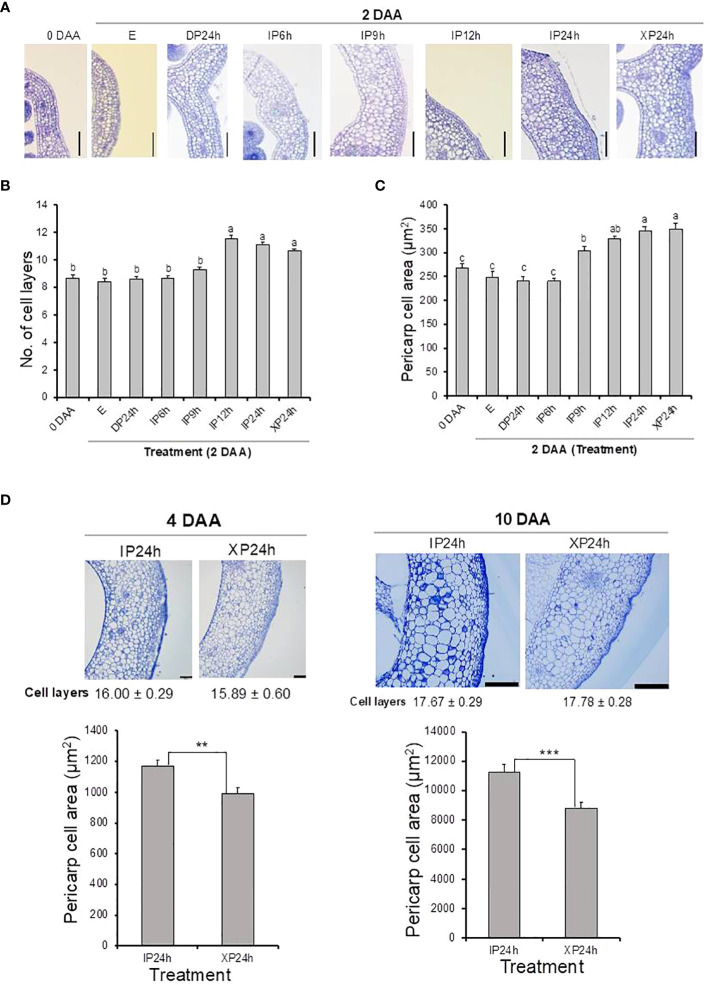
Histological analysis of ovary pericarp tissues after pollination with three different pollen types and style removal treatments. Ovaries were collected at 0 DAA and 2 DAA **(A–C)**, and at 4 DAA and 10 DAA **(D)**. Samples were stained with toluidine blue after carrying out the Technovit 7100 (Kulzer Technik, Germany) procedure, and observed under a light microscope at a magnification of 100X. Data in **(B, D)** are means ± SE. Different letters in **(B, C)** indicate statistical difference using Tukey’s HSD test at *P* < 0.05. Significant differences in **(D)** bar charts were analysed using Student’s t-test assessment (**P < 0.01; ***P < 0.001). White bars = 100 μm in **(A, D)** (4 DAA) and 500 μm in **(D)** (10 DAA). IP6h – pollinated with intact pollen and styles removed 6 h later; IP9h – pollinated with intact pollen and styles removed 9 h later; IP12h – pollinated with intact pollen and styles removed 12 h later; IP24h – pollinated with intact pollen and styles removed 24 h later; XP24h – pollinated with x-ray-irradiated pollen and styles removed 24 h later; DP24h – pollinated with dead pollen and styles removed 24 h later; (E) – emasculated but unpollinated control.

After fruit set assessments, we asked whether full penetration of pollen tubes into the ovaries minus fertilization can also lead to normal cell division and expansion. Histological analyses showed that both the number of cell layers and cell sizes of pericarp tissues of XP24h ovaries were higher at 2 DAA compared to the initial 0 DAA ovaries (**Figures 2A–C**). It is worth noting that both the number of cell layers and area of pericarp cells in XP24h ovaries at 2 DAA were not statistically different from those of IP12h and IP24h ovaries at the same timepoint (**Figures 2B, C**). By contrast, IP9h ovaries, which had only partial pollen tube penetration (**Figure 1A**), only showed larger cells at 2 DAA with no significant increase in cell layer numbers. Ovaries of IP6h, DP24h and the control E showed insignificant changes in both the cell area and number of cell layers, and the cell sizes were similar to those of ovaries at 0 DAA.

Pericarp cell measurements were also conducted on the young fruits (at 4 and 10 DAA) that developed from XP24h and IP24h ovaries. As shown in **Figure 2D**, young fruits of XP24h and IP24h showed a comparable number of cell layers both at 4 and 10 DAA. However, IP24h fruits showed a consistently larger pericarp cell area than XP24h fruits at the evaluated timepoints. It is worth noting that from 0 DAA to 2 DAA, there were no significant differences between IP24h and XP24h with regards to the increase in both cell layer numbers (26%) and cell area (30%) (**Figures 2A–C**). At 4 DAA, however, the increase in cell area relative to control E ovaries at 2 DAA was remarkably higher (210%) than that of the number of cell layers (46%). Furthermore, there was only a slight increase in cell layer numbers between 4 DAA and 10 DAA (11%), but a remarkably sharp increase in cell area (830%) was recorded. Altogether, these findings suggested that during early stages of fruit development (0–4 DAA), cell division (indicated by cell layer numbers) has a greater contribution than cell expansion (indicated by cell area). By contrast, cell expansion has a greater contribution than cell division during later fruit developmental stages (from 4 DAA).

**Figure 3:**
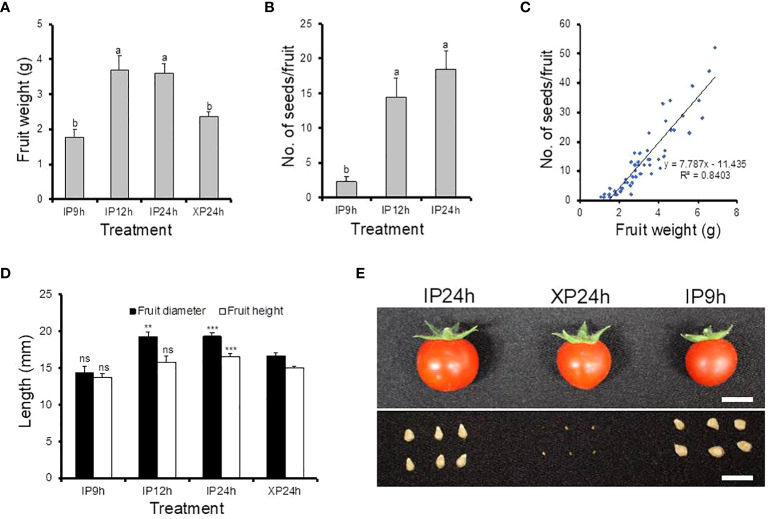
Morphological characteristics and seed production of fruits derived from IP9h, IP12h, IP24h and XP24h ovaries. **(A)** Average fruit weight. **(B)** Number of seeds per fruit. **(C)** The correlation between fruit weight and seed number. **(D)** Fruit diameter and height. Values in **(A, B, D)** are means ± SE of 10 fruits. Different letters in **(A, B)** indicate statistical difference, using Tukey’s HSD test at *P* < 0.05. Student’s T-test analysis was conducted to compare fruit indexes between XP24h and other treatments; *** = *P* < 0.001, **= *P* < 0.01, and ns = not significant. **(E)** Images showing the appearance of fruits and their respective seeds. White bars = 1 cm. IP9h – pollinated with intact pollen and styles removed 9 h later; IP12h – pollinated with intact pollen and styles removed 12 h later; IP24h – pollinated with intact pollen and styles removed 24 h later; XP24h – pollinated with x-ray-irradiated pollen and styles removed 24 h later.

To examine the impact of fertilization on late fruit developmental stages, fruit size and seed numbers in ripe fruits derived from style removal treatments that successfully set fruits (IP9h, IP12h, IP24h and XP24h) were also determined (**Figure 3**). The largest fruit weight was registered in ripe fruits obtained from IP12h and IP24h (3.69 g and 3.61 g, respectively) (**Figure 3A**). Fruits that developed from IP9h ovaries displayed the smallest average weight (1.77 g), but XP24h fruits had a slightly higher average weight (2.38 g), albeit significantly smaller than IP12h and IP24h fruits. Both the diameter and height of IP12h and IP24 h fruits were bigger than those of XP24h (**Figure 3D**), which correlated well with fruit weight data. These results suggested that the degree of fertilization might have a positive impact on the final size of fruits at maturity. Specifically, IP12h and IP24h ovaries underwent full penetration of pollen tubes (and hence presumably, complete fertilization) which likely led to the development of normal-sized fruits. However, IP9h ovaries underwent only partial pollen tube penetration, and hence partial fertilization, which might account for the small fruit sizes. Indeed, there was no significant difference in the number of seeds per fruit for IP12h and IP24h, but IP9h fruits had noticeably a smaller number of seeds (**Figure 3B**). Likewise, XP24h ovaries did not undergo fertilization even though full penetration of pollen tubes from the sterile X-ray-irradiated occurred, likely contributing to smaller fruit sizes than IP12h and IP24h. It is also worth noting that XP24h fruits did not produce regular seeds; instead, they produced many tiny seed-like structures (**Figure 3D**), which failed to germinate (data not shown). This finding therefore confirmed that the X-ray-treated pollen used in the present study indeed lost their fertilization function. Finally, combination of fruit weight and seed number data revealed a strong positive correlation (R^2 ^= 0.85) between these two phenotypes (**Figure 3C**).

### 3.3 Transcriptome sequencing analysis

**Figure 4:**
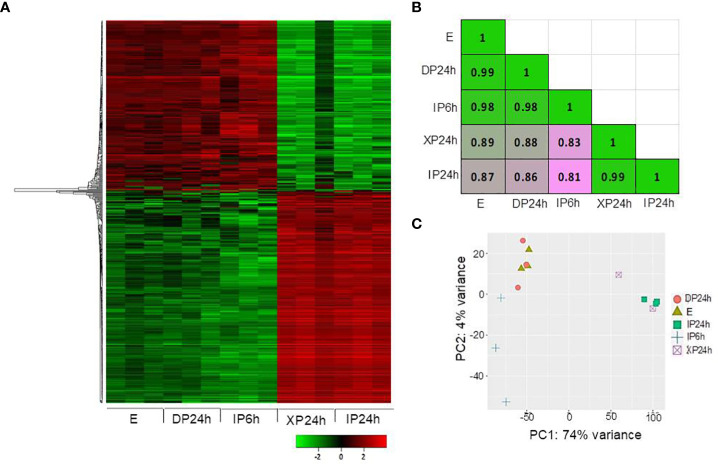
Overall expression patterns of the top variable genes. **(A)** Heatmap based on the log_10_ transformed TPM values of the top 500 variable genes. **(B)** Pearson’s correlation coefficient analysis using 75% of the top variable genes. **(C)** Principal component analysis (PCA) results showing PC1 and PC2. The ovary samples were collected 48 h after pollination (2 DAA), and each sample contained 5 ovaries with three replications. IP6h – pollinated with intact pollen and styles removed 6 h later; IP24h – pollinated with intact pollen and styles removed 24 h later; XP24h – pollinated with x-ray-irradiated pollen and styles removed 24 h later; DP24h – pollinated with dead pollen and styles removed 24 h later; E – emasculated but unpollinated control.

After phenotypical analyses, RNA-Seq was performed to identify transcriptomic changes in ovaries at 2 DAA triggered by the different pollen types and style removal times. A total of 500 most variable genes were used to construct a heatmap showing the overall expression patterns based on the log_10_ transformed TPM values. As shown in **Figure 4A**, the expression patterns in DP24h and IP6h ovaries were similar to those in the control (E) ovaries, while XP24h and IP24h ovaries showed highly similar patterns. Indeed, Pearson’s correlation coefficient analysis, based on 75% of the top variable genes, confirmed that there were highly positive correlations between XP24h and IP24h samples (r = 0.99), while there were high correlations (r=0.98~0.99) among E, DP24h, and IP6h samples (**Figure 4B**). In addition, principal component analysis (PCA) was conducted to explain 78% of the variation in the datasets (PC1 = 74%, and PC2 = 4%), in which XP24h and IP24h samples were also grouped together, but clearly separated from E, DP24h, and IP6h (**Figure 4C**).

### 3.4 Differential gene expression analysis

**Figure 5:**
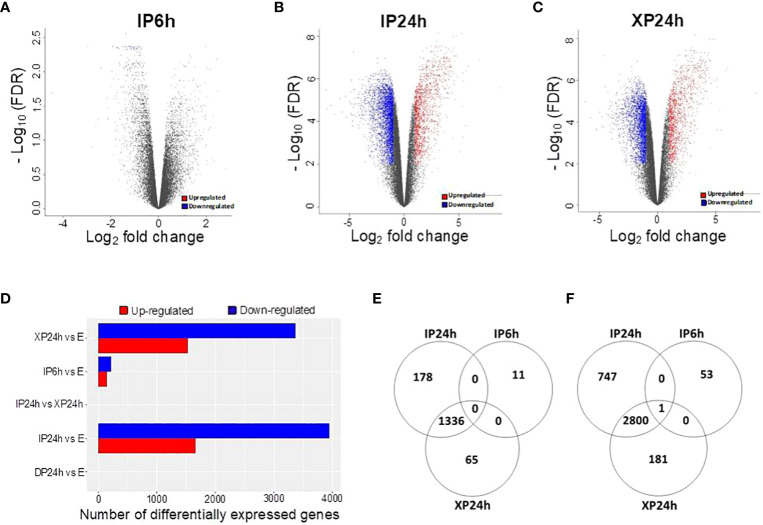
Identification of differentially expressed genes (DEGs) and the effect of pollen type and timing of style removal on their expression. **(A–C)** Volcano plots of all significant DEGs (log_2_FC ≥ 1 and - log_10_FDR ≥ 2) for IP6h, IP24h, and XP24h ovaries. Grey dots represent genes with insignificant expression changes; blue and red dots represent down-regulated and up-regulated genes, respectively. **(D)** Number of DEGs for the indicated treatment comparisons. Venn diagrams showing common or unique up-regulated **(E)** and down-regulated **(F)** genes relative to the control **(E)** samples. IP6h – pollinated with intact pollen and styles removed 6 h later; IP24h – pollinated with intact pollen and styles removed 24 h later; XP24h – pollinated with x-ray-irradiated pollen and styles removed 24 h later; DP24h – pollinated with dead pollen and styles removed 24 h later; E – emasculated but unpollinated control.

The DEGs with a minimal fold change of 2 and FDR < 0.01 were considered significant and selected for enrichment analysis. As illustrated in **Figures 5A, D**, 65 DEGs were detected in IP6h ovaries, of which 11were upregulated while 54 were downregulated compared to the control (E). The numbers of DEGs in XP24h ovaries were 4383 (1401 up-regulated and 2982 down-regulated) (**Figures 5C, D**), while in IP24h ovaries, 5062 DEGs (1514 up-regulated and 3548 down-regulated) were detected (**Figures 5B, D**). By contrast, there were no DEGs between DP24h vs E samples or between XP24h vs IP24h (**Figure 5D**).

Venn diagrams were also constructed to visualize the commonly or uniquely up- or down-regulated DEGs under different pollen treatments. This analysis revealed that XP24h and IP24h samples shared the majority of up-regulated DEGs (1336 genes), while there were 11, 65, and 178 uniquely up-regulated genes in IP6h, XP24h, and IP24h samples, respectively (**Figure 5E**). Among the downregulated DEGs, 2800 genes were common between XP24h and IP24h samples (**Figure 5F**). We found that 53, 181, and 747 genes were uniquely downregulated in IP6h, XP24h and IP24h samples, but only 1 gene was commonly downregulated in all three samples (**Figure 5F**).

### 3.5 DEG enrichment analysis

**Figure 6:**
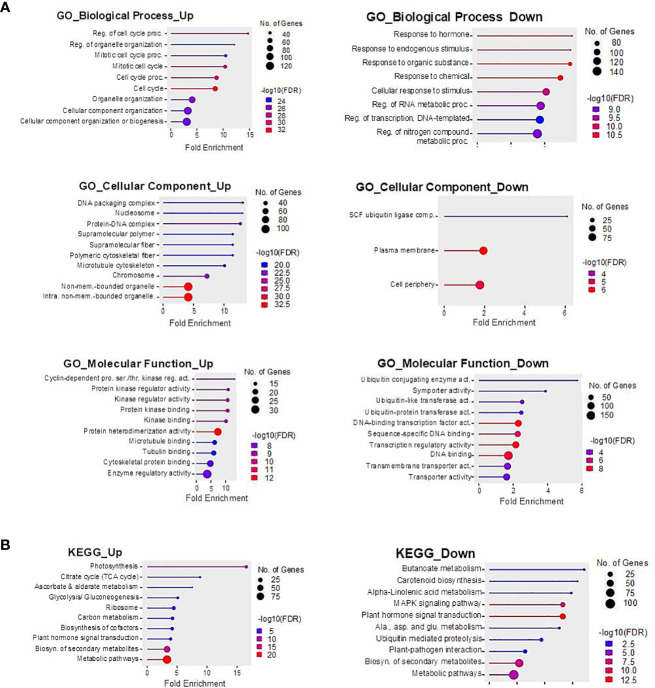
Gene Ontology **(A)** and KEGG pathways **(B)** enriched among up-regulated and down-regulated DEGs in XP24h ovaries. The GO terms and KEGG pathways were selected based on FDR values and sorted by fold enrichment values. The size of the dots represents the number of genes, and different colours indicate -log_10_(FDR) values.

Term enrichment analysis of the identified DEGs was then performed in an attempt to zoom in on the molecular changes triggered in XP24h ovaries relative to E ovaries (**Figure 6**). We found that the 1,401 up-regulated genes were classified into 40 GO terms and 15 KEGG pathways (**Table S1**). The top biological processes were ‘regulation of cell cycle process’ (30 genes), ‘mitotic cell cycle’ (44 genes), ‘cell cycle process’ (52 genes), and ‘cell cycle’ (61 genes) (**Figure 6A**). Additionally, the tope enriched cellular components were ‘DNA packing complex’ (28 genes), ‘nucleosome’ (27 genes), and ‘protein−DNA complex’ (32 genes), while molecular functions that stood out were ‘cyclin-dependent protein serine/threonine kinase regulator activity’ (13 genes), ‘protein kinase regulator activity’ (14 genes), ‘kinase regulator activity’ (15 genes), and ‘protein kinase binding’ (15 genes). For the KEGG pathways, 15 terms were found among the upregulated genes and these were related to many critical pathways such as ‘photosynthesis’ (13 genes), ‘biosynthesis of cofactor’ (15 genes), ‘plant hormone signal transduction’ (13 genes), and ‘biosynthesis of secondary metabolites’ (57 genes) (**Figure 6B**; **Table S1**).

The 2,982 downregulated DEGs belonged to 32 GO terms and 10 KEGG pathways (**Table S1**). Notable GO terms included ‘response to hormone’ (63 genes), ‘response to endogenous stimulus’ (63 genes), ‘response to organic substance’ (72 genes), ‘response to chemical’ (86 genes), ‘SCF ubiquitin ligase complex’ (9 genes), ‘plasma membrane’ (86 genes), ‘cell periphery’ (99 genes), ‘ubiquitin conjugating enzyme activity’ (9 genes), ‘DNA-binding transcription factor activity’ (86 genes), and ‘transcription regulator activity’ (91 genes) (**Figure 6A**). Furthermore, the main enriched KEGG pathways were ‘plant hormone signal transduction’ (34 genes), ‘plant−pathogen interaction’ (14 genes), and ‘biosynthesis of secondary metabolites’ (72 genes) (**Figure 6B**; **Table S1**).

Interestingly, the term enrichment analysis results for IP24h ovaries were highly similar to those of the XP24h ovaries (**Figure S1B**; **Table S1**). In contrast, the GO terms and KEGG pathways for the 65 DEGs (11 up-regulated and 54 downregulated) identified in IP6h ovaries were different. Notable GO terms and KEGG pathways in IP6h ovaries were ‘deoxyribonucleotide biosynthesis process’ ‘nucleosome assembly’, ‘chromatin remodelling’, ‘nucleosome’, ‘DNA packing complex’, ‘asparagine synthase (glutamine-hydrolysing) activity’, ‘purine metabolism’ and ‘pyrimidine metabolism’ (**Figure S1A**; **Table S1**). These findings further supported the notion that partial pollen tube might have limited effects on the remodelling of genetic materials. The changes induced might be essential preparations for cell division, but they are likely not sufficient to initiate fruit set in tomato.

### 3.6 Identification of potential genes/gene families responding to fully elongated pollen tubes

**Figure 7:**
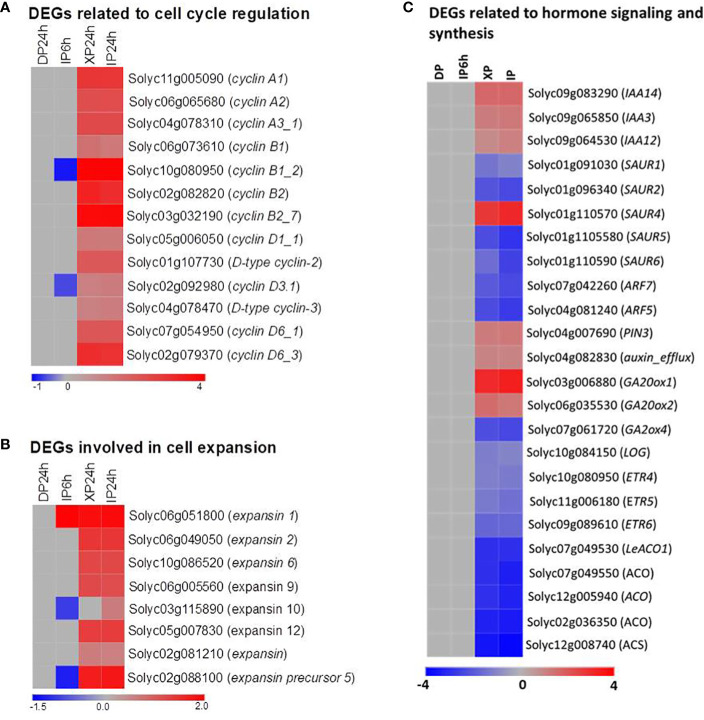
Expression patterns of genes involved in cell division and expansion, and phytohormones. **(A)** DEGs belonging to the cyclin family. **(B)** DEGs belonging to the expansin family. **(C)** DEGs related to phytohormone signalling, synthesis, and transportation. IP6h – pollinated with intact pollen and styles removed 6 h later; IP24h – pollinated with intact pollen and styles removed 24 h later; XP24h – pollinated with x-ray-irradiated pollen and styles removed 24 h later; DP24h – pollinated with dead pollen and styles removed 24 h later.

To explain the histological results that XP24h led to increased cell layer numbers and cell size at 2 DAA, we examined the expression patterns of some genes which are well-known for regulating cell division and expansion at the early stage of tomato fruit development. As illustrated in **Figure 7A**, thirteen members belonging to three subclasses (A, B, and D) of the cyclin family were significantly upregulated in XP24h and IP24h ovaries; highly expressed genes were *cyclin B1_2* (Solyc10g080950) and *cyclin B2_7* (Solyc03go32190). Likewise, five members of the expansin gene family and one gene encoding an expansin precursor (Soly02g088100) showed up to 3.6-fold higher expression in XP24h and IP24h ovaries (**Figure 7B**), than in IP6h and DP24h ovaries.

The vital role of phytohormones in regulating tomato fruit initiation and development was illustrated consistently through previous reports (Fenn and Giovannoni, 2021). In the present study, the KEGG enrichment analysis results also revealed that many DEGs were involved in the ‘plant hormone signal transduction’ pathway (**Figure 6B**). We therefore examined the expression pattern of genes involved in the synthesis, transportation, and signalling of various hormones (**Figure 7C**; **Table S2**). As indicated in **Figure 7C**, ethylene and auxin seemed to be the most active hormones in XP24h and IP24h ovaries. The expression of 8 genes related to ethylene (3 encoding ethylene receptors and 5 encoding ethylene biosynthesis enzymes) and 12 genes related to auxin (10 involved in auxin response and signalling, and 2 involved in auxin transport) were changed significantly. Specifically, there was significant downregulation (log_2_FC = -2) of *ARF7* and *ARF5*, which are key transcription factors that regulate fruit set and early fruit development. In addition, XP24h and IP24h ovaries displayed increased expression of two genes that positively regulate gibberellin synthesis (*GA20ox1* and *GA20ox2*), and repression of *GA2ox4*, a negative controller of gibberellin catabolism (**Figure 7C**). Altogether, these results illustrated that complete penetration of pollen tubes (as in XP24h) might broadly affect hormonal responses to activate both cell division and cell expansion events by increasing the expression of cyclin and expansin genes.

### 3.7 The effect of fully elongated pollen tubes on the expression of genes associated with parthenocarpy

**Figure 8:**
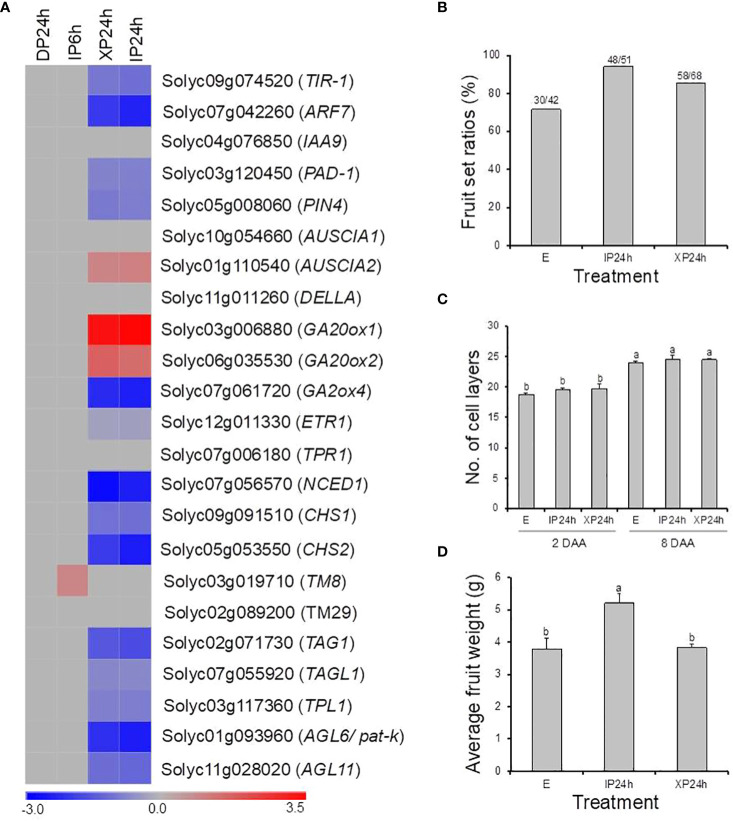
The potential involvement of well-known parthenocarpic genes in seedless fruit formation in flowers pollinated with X-ray irradiated pollen. **(A)** The expression patterns of 21 genes (previously associated with parthenocarpy in tomato) as affected by the different pollen types and style removal timing. **(B–D)** The effect of pollination with IP or XP and varying style removal timings on fruit initiation and development in *iaa9-3*, a well-established parthenocarpic “Micro-Tom” mutant line. Different letters in C and D indicate statistical difference, using Tukey’s HSD test at *P* < 0.05. IP24h – pollinated with intact pollen and styles removed 24 h later; XP24h – pollinated with x-ray-irradiated pollen and styles removed 24 h later; E – emasculated but unpollinated control.

In this study, pollination by X-ray-irradiated pollen resulted in a considerably high fruit set ratio (78.3%) of parthenocarpic fruits (**Figure 1B**), with an average weight approximately 65% that of seeded fruits on the same plant (**Figure 3A**). These effects are comparable to many previously reported parthenocarpy mutations in tomato (Sharif et al., 2022). We therefore examined the expression patterns of 23 well-known parthenocarpic genes in XP24h ovaries, compared to IP6h, IP24h, and DP24h. As a result, we found that 10 of these genes were differentially expressed (log_2_FC ≥ 2), particularly in XP24h and IP24h ovaries (**Figure 8A**). The top variable genes were *GA20ox1* (log_2_FC = 3.09), *NCED1* (log_2_FC = -2.75), *GA2ox2* (log_2_FC = -2.26), *AGL6* (*pat-k*) (log_2_FC = -2.17), and *ARF7* (log_2_FC = -2.02).

Although many genes related to auxin signalling and transport were differentially expressed in XP24h ovaries, *IAA9* which is an early auxin-responsive gene remained unchanged (**Figure 8A**). To further examine whether IAA9 is involved in parthenocarpic fruit development from XP24h ovaries, we pollinated the flowers of *iaa9-3*, a well-known parthenocarpic mutant (Saito et al., 2011), with X-ray-irradiated pollen. Compared to the control (E), X-ray-irradiated pollen slightly increased fruit set by approximately 14% (**Figure 8B**), but had little effect on cell layer formation (**Figure 8C**). In addition, the average weight of XP24h fruits (3.82 g) was similar to that of E fruits (**Figure 8D**), both of which were nearly 75% of the weight of IP24h fruits. These results suggested that full pollen tube penetration might regulate auxin signalling *via* altering the interaction between ARF7 and IAA9 proteins.

## 4 Discussion

The role of pollen tubes in both the production of seedless fruits and study of parthenocarpic mechanisms has been well established in watermelons, through pollination tests with soft X-ray-irradiated pollen (Sugiyama and Morishita, 2002; Qu et al., 2016; Hu et al., 2019), or foreign (bottle gourd) pollen (Sugiyama et al., 2014). In either case, penetration of watermelon ovaries by pollen tubes led to reasonably high fruit set and the resultant parthenocarpic fruits had virtually the same size as control seeded fruits, most likely due to accumulation of various phytohormones including auxins, gibberellins, and cytokinins (Hu et al., 2019). Attempts to replicate these findings in tomato (both *S. lycopersicum* and *S. pimpinellifolium*) have been unsuccessful to date, as irradiation with X-ray or gamma-ray on dried pollen severely affected pollen germination, reduced fruit set, and resulted in very tiny parthenocarpic fruits (Nishiyama and Tsukuda, 1961; Uematsu and Nishiyama, 1967). In the present study, X-ray irradiation was applied on fresh anther cones before drying, as opposed to previous studies where it was applied directly on dried pollen. As a result, we found that pollen germination was unaffected and pollen tubes from XP elongated in a similar manner as those from IP, fully penetrating the ovaries 24 h after pollination (**Figure 1A**). This observation allowed us to further study the distinct function of pollen tubes in tomato fruit formation.

It is intriguing that full penetration of the ovaries by pollen tubes emanating from XP triggered comparably high fruit set (**Figure 1B**), unlike partial pollen tube growth (as in IP6h) which resulted in failed fruit set. Although the parthenocarpic fruit which developed after XP pollination were slightly smaller than seeded fruit obtained by IP pollination (**Figure 3**), their average weight was within acceptable limits of most previously reported parthenocarpic tomato fruits. A possible explanation for the relatively small-sized XP-derived fruits is the lack of fertilization, evidenced by production of empty seeds (**Figure 3E**). In strawberry, successful fertilization was recently shown to induce auxin biosynthesis, resulting in normal fruit growth and development (Guo et al., 2022). Increased expression of auxin biosynthetic genes coupled with accumulation of auxins was also reported in watermelon ovaries at 2 DAA following pollination with X-ray-irradiated pollen (Hu et al., 2019), which most likely accounted for comparable sizes between parthenocarpic and seeded fruits (Sugiyama and Morishita, 2000; Sugiyama and Morishita, 2002). In the present study, auxins were not quantified but transcriptome analysis revealed that only auxin signalling and transportation genes were differentially expressed while auxin biosynthetic genes remained unchanged in XP-pollinated ovaries at 2 DAA (**Figure 7C**). Given that the genes associated with cell expansion were upregulated in XP-pollinated ovaries to a similar degree as IP-pollinated ones (**Figure 3E**), it is plausible that lack of fertilization (and hence no auxin accumulation) hinders normal fruit growth and development. The auxin effect most likely targets cell expansion rather than cell division (**Figure 2D**), ultimately affecting fruit size at maturity. This hypothesis is further supported by recent findings that the auxin content in ovaries of a new parthenocarpic tomato line “R35-P” was twice as much as that of the normal line “R35-N” (Zhang et al., 2021), resulting in similar-sized seeded and seedless fruits. Future studies should explore possible strategies to increase auxin content at early developmental stages for production of normal-sized parthenocarpic tomatoes.

In the present study, many previously reported parthenocarpic genes were differentially expressed in response to ovary penetration by pollen tubes (**Figure 8A**), which was consistent with their respective mutants (Schijlen et al., 2007; De Jong et al., 2009; García-Hurtado et al., 2012; Martínez-Bello et al., 2015; Liu et al., 2018; Takisawa et al., 2018). This finding suggests that the regulatory mechanisms by which full pollen tube penetration into the ovary induces seedless fruit development in tomato involves the coordinated action of diverse parthenocarpic genes. However, it was puzzling that the expression of *IAA9* did not change (**Figure 8A**), yet its loss-of-function mutant *iaa9-3* was shown to induce parthenocarpy (Saito et al., 2011). Pollination of *iaa9-3* flowers with XP slightly increased fruit set but failed to impact fruit development (**Figures 8B–D**). A possible explanation lies in the downregulation of *ARF7* following pollination of wild type “Micro-Tom” flowers with XP (**Figure 8A**). Previously, the ARF7/IAA9 complex was shown to regulate fruit initiation in tomato by acting as a transcriptional repressor of auxin and gibberellin biosynthetic genes (Hu et al., 2018). Therefore, a decrease in *ARF7* transcript levels points towards a weakening ARF7/IAA9 complex, which would then result in increased expression of gibberellin biosynthetic genes (**Figure 7C**), as well as *expansins* and *cyclins* (**Figures 7A, B**), eventually leading to fruit initiation and development.

Both ethylene production and signalling were also reported to change significantly during pollen tube growth in tomato (Althiab-Almasaud et al., 2021), and increased ethylene content in young tomato ovaries inhibit fruit set, either by promoting pedicel abscission (Roberts et al., 1984), or inhibiting cell division (Vandenbussche et al., 2007). In the present study, ethylene biosynthetic genes were down-regulated in ovaries at 2 DAA following full penetration by pollen tubes from XP (**Figure 7C**), a change that, in all likelihood, would avert pedicel abscission and promote cell division culminating in successful fruit formation.

**Figure 9:**
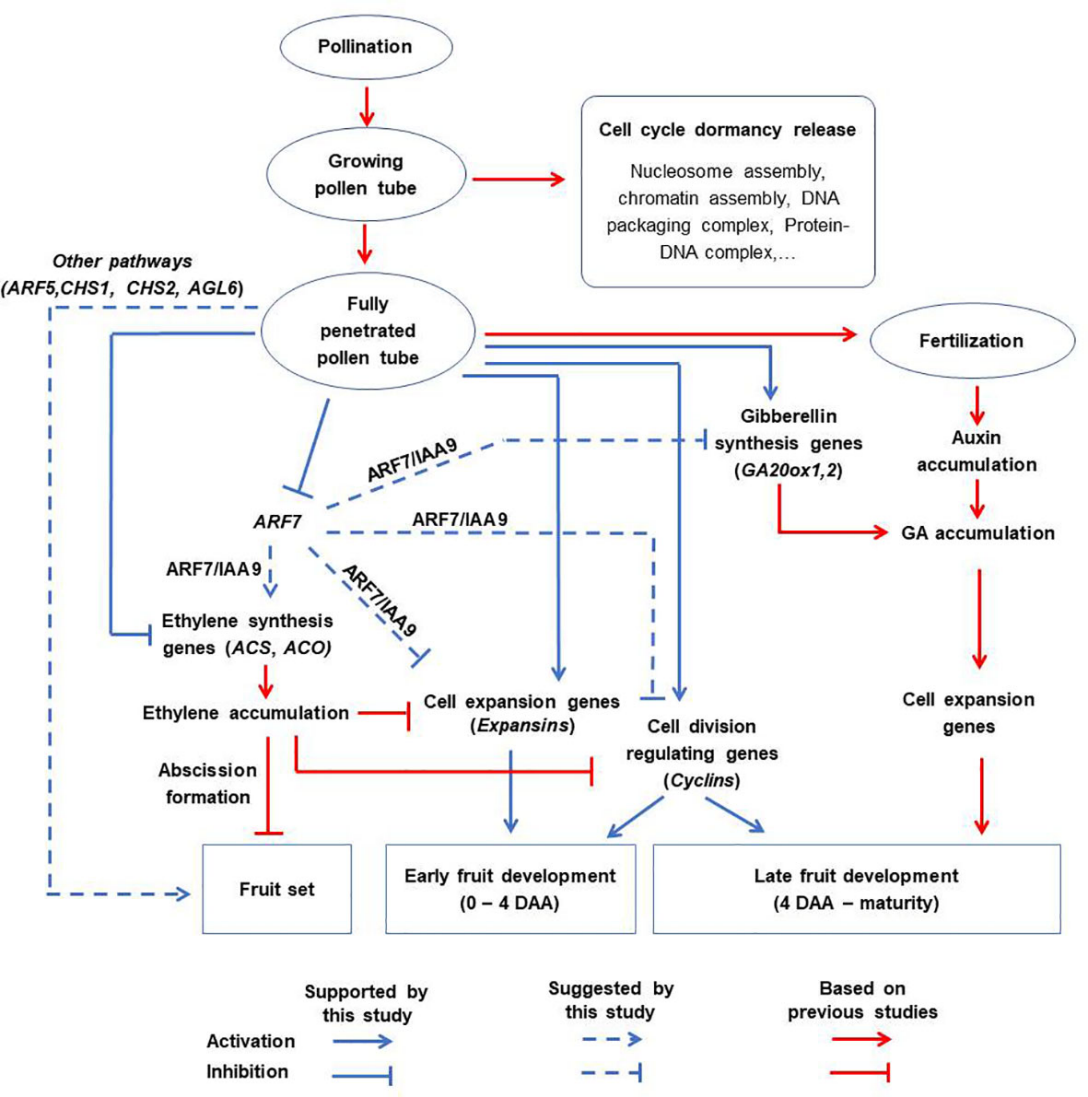
A proposed schematic model for tomato fruit initiation and development. Pollination alone has no significant effect. Partial pollen tube growth alters the expression of genes related to cell cycle dormancy release but this is not sufficient to initiate fruit set. Full penetration of pollen tubes into ovaries initiates fruit set and contributes to the early stages of fruit development (up to 4 DAA) *via* diverse pathways which might include inhibition of ethylene biosynthetic genes, stimulation of genes associated with gibberellin biosynthesis (*GA20ox1*), cell expansion (*expansins*), and division (*cyclins*). This might involve diverse parthenocarpic genes (particularly *ARF7*) as intermediate regulators. Finally, fertilization is responsible for auxin accumulation, which enhances the expression of cell expansion genes and hence contributes to late fruit development.

Based on the results of this study, we propose a revised schematic model illustrating the distinct contributions of pollination, pollen tubes and fertilization to fruit formation in tomato (**Figure 9**). According to this model, the effect of pollination alone is non-significant. Partial pollen tube growth (or growing pollen tubes) significantly alters several pathways related with ‘nucleosome assembly’, ‘chromatin assembly’, ‘DNA packaging complex’, and ‘protein-DNA complex’, to release cell cycle dormancy. However, these changes are limited and cannot sufficiently activate cell division and expansion processes, resulting in failed fruit set. On the other hand, full pollen tube penetration sufficiently initiates fruit set and contributes to the early stages of fruit development (at least up to 4 DAA) by regulating different hormonal pathways likely through diverse parthenocarpic genes, such as *ARF7*, *ARF5*, *CHS1*, *CHS2*, *AGL6*, and *GA20ox1*. Finally, fertilization contributes to fruit development primarily by upregulating auxin synthesis, which then stimulates gibberellin synthesis and accelerates the expression of late-responding cell expansion genes. The effect of fertilization is evident towards later stages (beyond 4 DAA) of fruit development. In addition, our results suggest that the contribution of cell expansion to fruit development is greater than that of cell division from 4 DAA onwards, which is a much earlier timepoint than previous models (Ariizumi et al., 2013; Quinet et al., 2019).

## 5 Conclusion

Overall, we reported that the complete penetration of pollen tubes into ovaries regulates the expression of essential genes involved in diverse pathways to accelerate both cell division and cell expansion events in tomato ovary tissue. These effects are independent of fertilization, resulting in a high fruit set ratio and parthenocarpic fruit development. These findings contribute to a better understanding of the mechanism by which tomato fruits are set and developed.

## Data availability statement

The datasets presented in this study can be found in online repositories. The names of the repository/repositories and accession number(s) can be found below: DNA Data Bank of Japan (DDBJ) database (DRR461225-DRR461239).

## Author contributions

All authors contributed to the article and approved the submitted version. Conceptualization: LT, HE, KS. Experimental design: LT, HE, KS. Data curation: LT, MK. Data analysis and visualization: LT, KS, OM. Writing-original draft preparation: LT, KS. Writing-review and editing: LT, HE, KS, OM, MK. Supervision: HE, KS. Resources: HE.

## Footnotes

1
http://tomatoma.nbrp.jp

2
http://usegalaxy.org

3
http://bioinformatics.sdstate.edu/idep93

4
http://bioinformatics.sdstate.edu/go
