# Supplementary figures and images for: Pollination, pollen tube growth, and fertilization independently contribute to fruit set and development in tomato
Source: Front Plant Sci. 2023 Jun 20;14:1205816. doi: 10.3389/fpls.2023.1205816 (PMC10319911; doi:10.3389/fpls.2023.1205816)

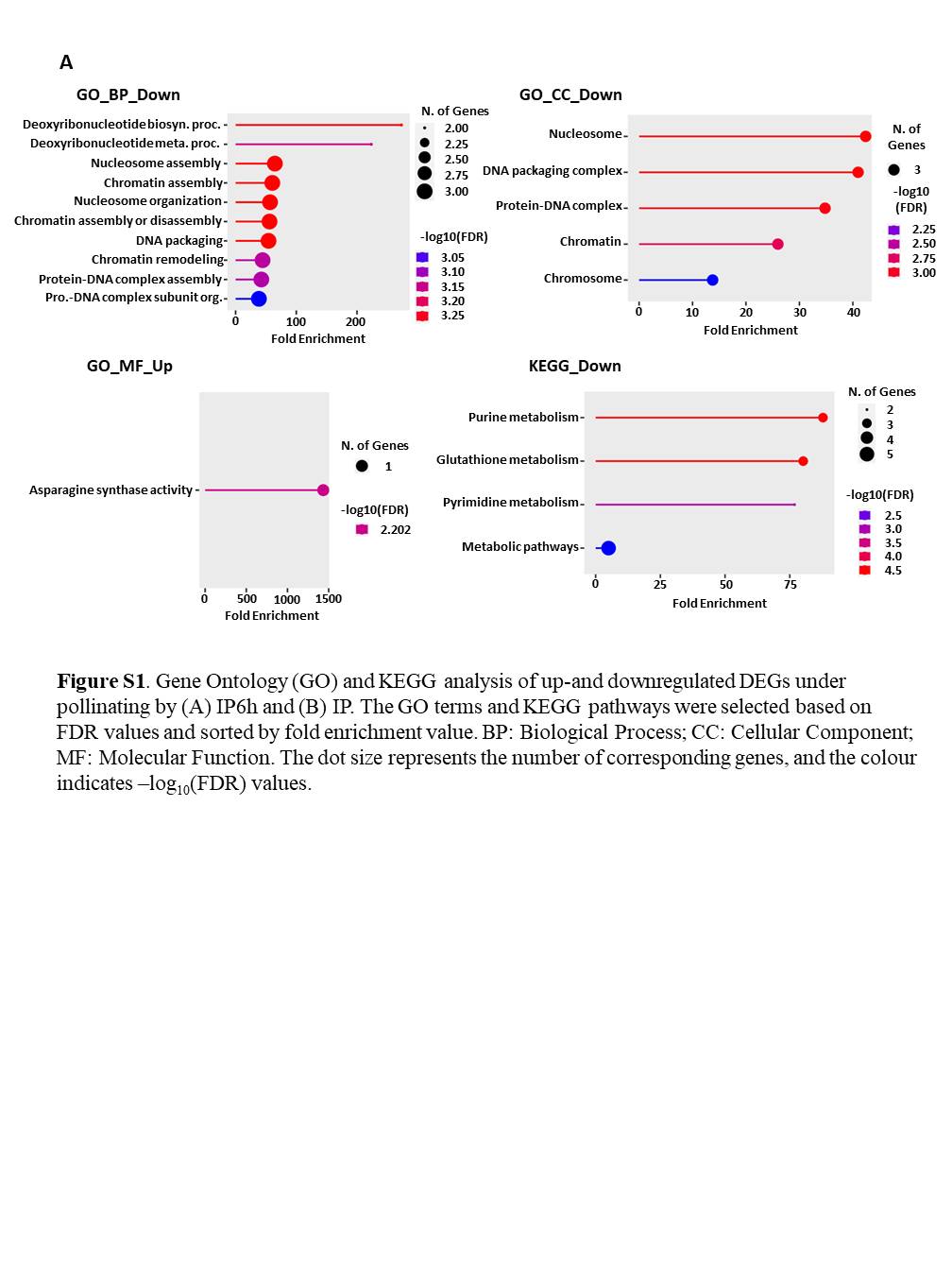

Supplement: Supplementary file 1 [file DataSheet_1.zip › Supplementary/Figure S1A.JPG]

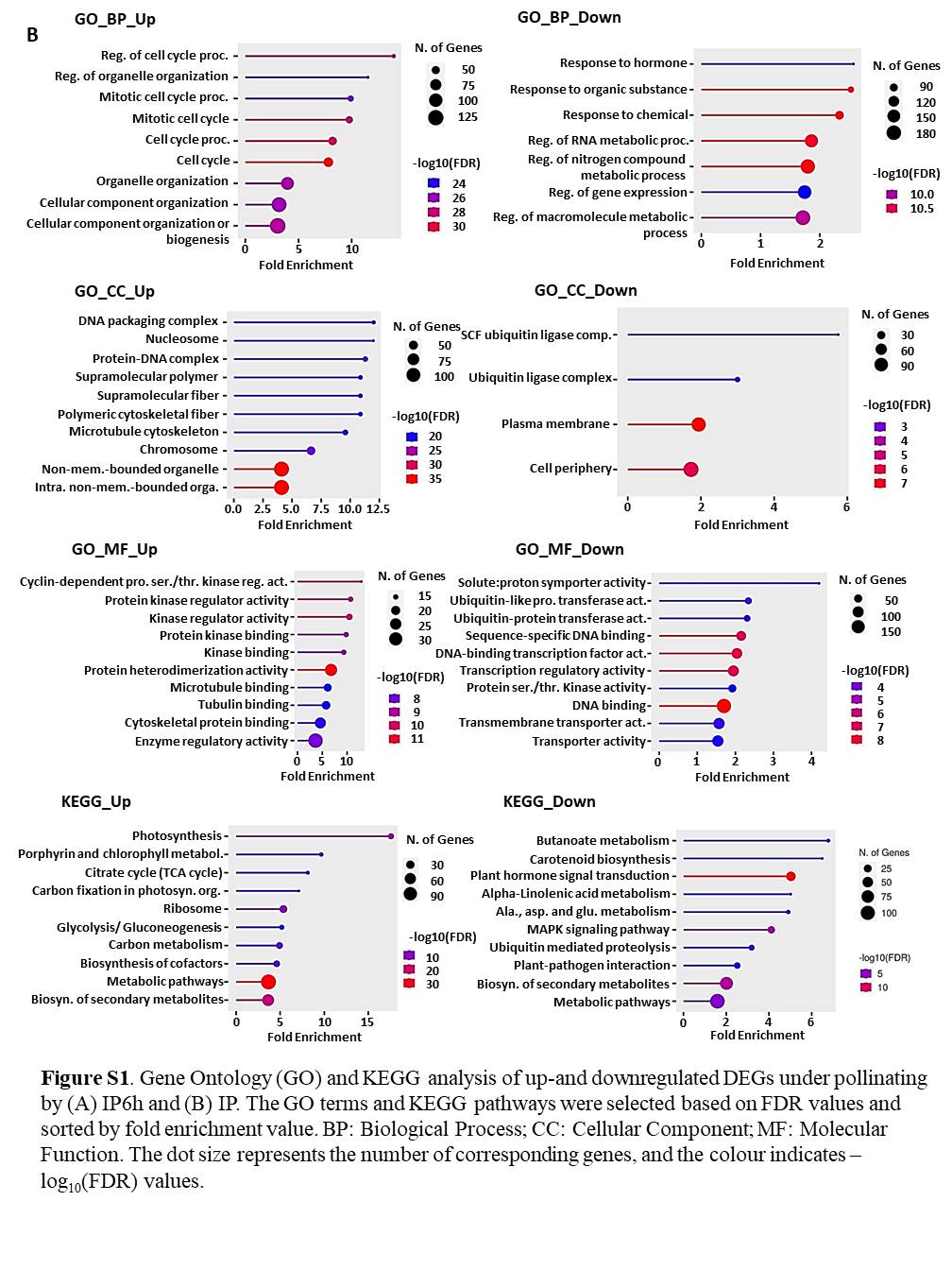

Supplement: Supplementary file 1 [file DataSheet_1.zip › Supplementary/Figure S1B.JPG]
